# Corn Response as Affected by Planting Distance from the Center of Strip-Till Fertilized Rows

**DOI:** 10.3389/fpls.2016.01232

**Published:** 2016-08-18

**Authors:** Eric Adee, Fernando D. Hansel, Dorivar A. Ruiz Diaz, Keith Janssen

**Affiliations:** Department of Agronomy, Kansas State UniversityManhattan, KS, USA

**Keywords:** strip-till, corn, *Zea mays*, nutrient uptake, stand establishment, grain yield

## Abstract

Strip-till has been used at a large scale in east central Kansas as an alternative to earlier planting dates under a no-till system. To determine the effects of planting corn (*Zea mays*) under previously established strip-tilled fertilized rows, experiments were conducted on an Osage silty clay loam soil in 2006 and 2008 and on a Woodson silt loam soil in 2009, 2010, and 2011 using three different planting distances from the strip-tilled fertilized rows (0, 10, 20, and 38 cm) with a strip-till operation performed between 1 and 73 days before planting. The depth of the strip-till fertilizer application was 13–15 cm below the soil surface. Corn that was planted 10 cm from the fertilized row showed greater early season growth, higher plant population, and grain yield. Planting 20 and 38 cm from the center of the fertilized rows showed none of the benefits that are typically associated with strip-tillage system. Enough time should be allowed between the strip-till operation and planting to reach satisfactory soil conditions (e.g., moist and firm seedbed). Our results suggest that the best location for planting strip-tilled fertilized corn vary depending on soil and climatic conditions as well as the time between fertilizer application with the strip-till operation and planting. With fewer number of days, planting directly on the center of fertilized strip-till resulted in decreased plant population and lower grain yield. However, the greatest yield benefit across different planting conditions was attained when planting within 10 cm of the strip.

## Introduction

The ability to strip-till for corn production in east central Kansas has allowed farmers to address several challenges they have faced. With the clay pan soils that are susceptible to erosion because of limited water infiltration, no-till has offered protection against soil erosion (Unger and Vigil, 1998; Lamm et al., 2009). While the layer of crop residue has offered protection against erosion, it has increased soil moisture and reduced soil temperatures, thus limiting the opportunities to plant corn early (Vetsch and Randall, 2002; Perez-Bidegain et al., 2007).

Earlier planting dates for corn have been shown to improve yields by getting the corn farther along in the growing season, especially to reach pollination, before the hot/dry weather of July (Roozeboom et al., 2009). Strip-till allows for residue to remain over much of the field to protect against erosion (Vetsch and Randall, 2002), while allowing the soil to warm and dry in the planting zone. The strip-till method also allows for banding of fertilizers to save a trip across the field and to place fertilizers closer to the plants for more efficient utilization (Fernandez and White, 2012). Fall application of fertilizer is not always possible or advisable due to either wet soil or soil temperatures that are too high to avoid de-nitrification of the applied N. With spring timing for the application of strips with fertilizer there can be challenges of getting the strips applied early enough so they have time to settle and make a good seedbed without air pockets. Corn stands and yields can be reduced in strip-till compared to conventional tillage (Hallauer and Colvin, 1985).

The increased use of automatic guidance systems technology (GPS and auto-steer), provide the opportunity for precise placement of corn seed related to previously established strip-tilled fertilized rows (Fernandez and Schaefer, 2011). The best placement of the seed at planting related to the strip-till area can vary depending on several factors, including the amount of time that has elapsed between the strip-tilled operation and planting as well as the rate and forms of fertilizers used. Therefore, planting directly on top of the strip till fertilized row may not be the best option. For example, freshly strip-tilled fertilized rows could be loose and have air pockets under the row (Hakansson et al., 2002), might be dry or cloddy (Cresswell et al., 1993), or could contain high levels of fertilizer that can generate seedling damage due to salts or ammonia (Fernandez and Schaefer, 2011). However, planting too far away from the strip-tilled fertilized rows might reduce the benefits of this tillage system (warmer soil, cleared residue, and rapid fertilizer-root contact). The objective of this study was to evaluate the effect of planting distance from the center of strip-tilled fertilized rows for corn production.

## Materials and methods

Field experiments were conducted on an Osage silty clay loam soil (Fine, smectitic, thermic Typic Epiaquerts, on 0–1% slope, land capability class IIw) near Lane, Kansas (38°25′N 95°07′W), in 2006 and 2008, and on a Woodson silt loam soil (Fine, smectitic, thermic Abruptic Argiaquolls, on 0–1% slope, land capability class IIs) at the East Central Kansas Experiment Field near Ottawa, Kansas (38°32″N 95°14″W), in 2009, 2010, and 2011. Both locations receive an average annual precipitation of ~1017 mm (Kansas State Weather Data Library, 2012). Soil samples were collected at the 0- to 15-cm depth from the fields where the studies where located. Samples were analyzed for soil test P (STP) using the Mehlich-3 extraction, and for soil test K (STK) using the ammonium acetate extraction (Warncke and Brown, 1998). Soil pH was measured in a 1:1 water suspension, and total organic matter was determined by loss on ignition (Combs and Nathan, 1998). Soil texture was estimated by the hydrometer method (Model 6026Q20, Thomas Scientific; Bouyoucos, 1962). Cation exchange capacity (CEC) was estimated by summing the exchangeable acidity and the exchangeable bases (Warncke and Brown, 1998, Table 1).

**Table 1:** **Soil characterization at two locations, Lane, and East Central Kansas Experiment Field in Ottawa**.

**Years**	**Site**	**Soil series**	**pH**	**OM**	**STP^†^**	**STK**	**CEC**	**Sand**	**Clay**
				**(g Kg^−1^)**	**(mg Kg^−1^)**		**(meq/100g)**	**(g Kg^−1^)**	
2006 and 2008	Lane	Osage	6.7	39.2	19.8	234.0	24.8	80	360
2009–2011	Ottawa	Woodson	6.9	27.6	6.7	154.5	19.0	100	300

† *STP, soil test phosphorus; STK, soil test potassium (K)*.

The combination of strip-till and fertilizer application was performed 2–73 days before planting (Table 2). Fertilizer was applied at a standard rate (134-34-11 kg ha^−1^ of N, P, and K, respectively). The fertilizer source used for this study include urea, diammonium phosphate, and potassium chloride. The depth of the fertilizer application with the strip-till was 13–15 cm. The applicator was a Yetter (Yetter Mfg., Colchester, IL) pull caddy with Maverick Generation 2 openers and residue managers (model 2984) and a 5 cm mole knife, equipped with a Gandy Orbit Air model 623016 box and metering system (Gandy Co, Owatanna, MN).

**Table 2:** **Strip-till fertilizer application, planting, and harvest dates, and corn hybrids used in planting distance from strip-till row**.

	**Years**				
	**2006**	**2008**	**2009**	**2010**	**2011**
Strip-till date	April 8	April 5	March 6	April 13	April 13
Planting date	April 10	April 22	May 18	June 2	May 2
Days to plant^†^	2	17	73	50	19
Rain before planting (mm)^§^	0	51	227	237	39
Hybrid	Pioneer 35P17	Midland 428 BTLL	DeKalb 5044	Midland 436RR	Pioneer 35F40
Harvest date	Sept 7	Oct 1	Oct 5	Oct 4	–^‡^

† *Number of days between strip-till fertilizer application and planting*.

‡ *Not available*.

§ *Total rainfall after strip-till operation and before planting*.

Planting was with a White 6100 planter (AGCO, Duluth, GA). The planting distances evaluated were directly on top of the strip-tilled fertilized rows and ~10, 20, and 38 cm off the center of the rows. The experiment was designed as a randomized complete block with three to four replications. Individual plot size ranged from 3.04 m (4 rows × 76 cm) wide and 48 m long to 6.08 (8 rows × 76 cm) and 240 m long, depending on the field location and year.

The planting treatments were evaluated for effects on early season corn growth, nutrient uptake, plant population at harvest, and grain yield. Early growth assessments were made by collecting the above ground portion of 12 plants at growth stage V2 to V3 (Abendroth et al., 2011), and the above ground portion of six plants at the V7 to V8 growth stage (Abendroth et al., 2011) from each plot. Plant dry weight, and nutrient concentration (N, P, and K) were measured, and nutrient uptake calculated based on these two parameters. Plant samples were dried at 65°C in a forced-air oven, weighed, and ground to pass through a 2-mm sieve. Nutrient analysis was completed on digested plant materials using the sulfuric acid-hydrogen peroxide (H_2_SO_4_–H_2_O_2_) method. Nitrogen and phosphorus in the extract was determined with the Technicon AAII autoanalyzer, and potassium by Flame Atomic Absorption. Plant populations at harvest were measured by counting plants in 48–131 m of row, depending on the length of row each year. The center two rows of each plot were harvested with a plot combine, grain weight, and moisture were measured, and yields adjusted to 155 g kg^−1^ moisture.

Statistical analysis was completed with SAS 9.4 (SAS Institute, 2014) using the GLIMMIX procedure. Significance level was set at the *P* < 0.05 for mean separation and using the LINES procedure of GLIMMIX. Anova analysis was completed by site-year using blocks as random in the model; and across site-years and using blocks and site-years as random effect in the model. Our statistical analysis used site-year as random in the model given that this factor can represent a larger population with a probability distribution in addition to the exchangeability of its effect. Our discussion and conclusions put emphasis on the main treatment effects across 5 site-years and the potential applicability for producers in future years.

## Results and discussion

Total rainfall from January to September was lower than average for 2006 and 2011 (Table 3). The amount of rainfall after the strip till operation and planting was also lower for years 2006 (zero) and 2011 (39 mm; Table 2). Dry soil conditions can be associated with higher potential for seedling damage from the fertilizer application as well as poor soil condition for early plant establishment (Perez-Bidegain et al., 2007).

**Table 3:** **Monthly and 30 year average precipitation for East Central Kansas Experiment Field, Ottawa**.

**Month**	**2006**	**2008**	**2009**	**2010**	**2011**	**Average^†^**
	**mm**					
January	18	21	1	14	13	31
February	0	82	26	37	64	37
March	51	76	83	38	72	68
April	85	70	181	122	55	98
May	96	136	41	116	130	137
June	33	197	200	162	68	143
July	85	86	117	139	22	104
August	212	98	165	52	61	103
September	54	173	149	149	62	105
Total	633	939	961	829	547	826

† *30 Year Average (1981–2010)*.

Across site-year analysis of all the parameters evaluated in the study showed significant effect by planting distance from the center of fertilized strip till except for P and K concentration at the V7–V8 growth stage (Table 4). Above-ground corn biomass early in the season (V2–V3, and V7–V8) was affected by planting distance treatments for each year during the study, other parameters were statistically significant only for some years or across years (Table 4).

**Table 4:** **Significance of *F*-values for the fixed effects of planting distance from the center of strip-till row on corn growth, nutrient concentration, nutrient uptake, population, and grain yield**.

**Variable**	**Years**					
	**2006**	**2008**	**2009**	**2010**	**2011**	**Across years**
	**P > F**					
Plant dry weight V2–V3	0.030	0.001	0.018	–^†^	<0.001	<0.001
N concentration V2–V3	0.028	0.012	0.033	–	0.029	<0.001
P concentration V2–V3	0.304	0.027	0.005	–	0.243	0.001
K concentration V2–V3	0.790	<0.001	0.014	–	0.014	<0.001
N Uptake V2–V3	0.019	0.001	0.025	–	0.072	<0.001
P Uptake V2–V3	0.100	0.007	0.115	–	0.124	0.001
K Uptake V2–V3	0.031	0.001	0.018	–	0.039	<0.001
Plant dry weight V7–V8	0.651	0.001	0.008	0.002	0.001	<0.001
N concentration V7–V8	0.211	0.014	0.487	0.002	0.070	0.013
P concentration V7–V8	0.329	0.348	0.872	0.021	0.660	0.220
K concentration V7–V8	0.663	0.720	0.335	0.712	0.203	0.147
N Uptake V7–V8	0.909	0.001	0.016	0.005	0.217	<0.001
P Uptake V7–V8	0.867	0.001	0.014	0.009	0.179	<0.001
K Uptake V7–V8	0.995	0.002	0.011	0.172	0.101	<0.001
Plant population	0.046	0.054	0.834	<0.001	0.001	<0.001
Grain yield	0.047	0.008	0.338	0.127	0.365	0.002

† *Not available*.

**Figure 1:**
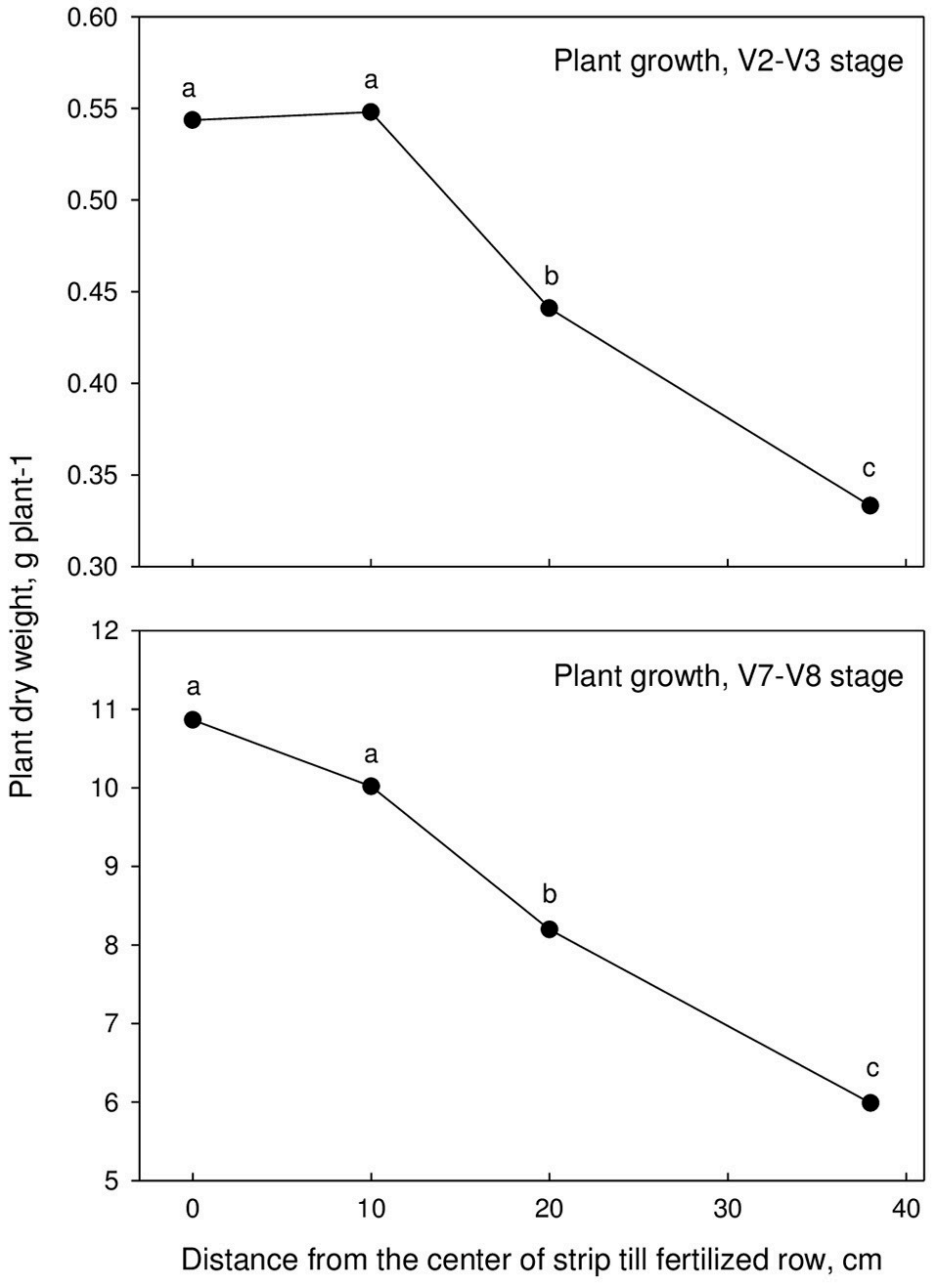
**Plant dry weight as affected by distance from the center of strip-till fertilized row across site-years**. Samples collected at to growth stage, V2–V3 and V7–V8.

Corn early growth at the V2–V3 and V7–V8 growth stages were generally higher for corn planted directly on top of the strip-tilled fertilized rows or within 10 cm (Tables 5, 6, Figure 1). Previous studies showed similar results where the beneficial effects of in-row tillage and residue removal in the seed-row zone with strip-till led to greater early growth of corn (Vetsch et al., 2007). Planting corn 20 cm from the center of the strip-tilled fertilized rows reduced early season growth of corn at the V7–V8 growth stage by 24% on average, and planting 38 cm away reduced early season growth by 40%. Cool conditions slow the germination of corn seed and slow shoot elongation before emergence influencing early season corn growth (Miedema and Sinnaeve, 1980).

**Table 5:** **Plant dry weight, nutrient concentration, and nutrient uptake at the V2–V3 growth stage^†^**.

**Distance^‡^**	**Plant dry weight**	**Nutrient concentration**			**Nutrient uptake**		
**cm**	**g plant^−1^**	**Nitrogen**	**Phosphorus**	**Potassium**	**Nitrogen**	**Phosphorus**	**Potassium**
		**g kg^−1^**			**mg plant^−1^**		
**2006**								
0	0.33b^§^	3.9a	0.26	2.1	13.0cb	0.88	7.0b
10	0.49a	3.9a	0.30	2.2	19.1a	1.47	10.7a
20	0.40ab	3.6ab	0.31	2.2	14.8ab	1.30	8.7ab
38	0.30b	3.0b	0.28	2.3	9.2c	0.87	6.8b
**2008**								
0	0.44ab	3.5a	0.33b	2.0a	15.4a	1.47ab	9.0a
10	0.47a	3.4a	0.33b	1.9b	15.8a	1.56a	9.0a
20	0.38b	3.3a	0.33b	1.6c	12.7b	1.27b	6.0b
38	0.23c	2.9b	0.42a	1.4d	6.6c	0.95c	3.1c
**2009**								
0	0.80a	4.0a	0.4b	2.3a	31.7a	3.17	18.9a
10	0.71a	4.0a	0.4b	2.2a	28.4a	2.85	15.7a
20	0.62ab	4.0a	0.44a	2.0a	25.5ab	2.73	12.9ab
38	0.46b	3.7b	0.46a	1.4b	17.0b	2.09	6.5b
**2011**								
0	0.56a	4.0a	0.41	2.3a	30.9a	3.17	18.4a
10	0.52b	4.0a	0.40	2.2a	28.4a	2.85	15.7a
20	0.37c	4.0a	0.44	2.0a	25.5ab	2.73	12.9ab
38	0.35c	3.7b	0.44	1.4b	17.8b	2.10	7.0b

† *Early growth data (V2–V3 growth stage) for 2010 was not collected*.

‡ *Distance from the center of strip till row*.

§ *Numbers followed by the same letter in a column within each year are not statistically significant at the 0.05 probability level*.

**Table 6:** **Plant dry weight, nutrient concentration, and nutrient uptake at the V7–V8 growth stage**.

**Distance^†^**	**Plant dry weight**	**Nutrient concentration**			**Nutrient uptake**			**Population**	**Grain yield**
**cm**	**g plant^−1^**	**Nitrogen**	**Phosphorus**	**Potassium**	**Nitrogen**	**Phosphorus**	**Potassium**	**Plants ha^−1^**	**Mg ha^−1^**
		**g kg^−1^**			**mg plant^−1^**				
**2006**									
0	6.5	4.3	0.48	1.9	279	30.9	124	58924b^‡^	8.2b
10	6.4	4.3	0.47	1.9	274	30.3	119	63015a	8.9a
20	6.6	4.2	0.46	1.8	281	30.2	121	63407a	8.5ab
38	5.7	4.6	0.50	2.1	262	28.2	119	59858ab	8.7ab
**2008**									
0	6.9a	2.8a	0.33	1.8	195a	22.7a	125ab	56128b	6.0cb
10	6.9a	2.8a	0.36	2.0	195a	24.5a	138a	57956a	6.5a
20	5.5a	2.8a	0.36	1.8	154b	19.9a	99b	55876b	6.3ab
38	2.4b	2.5b	0.33	1.9	59c	7.5b	44c	55805b	5.7c
**2009**									
0	19.5a	3.0	0.41	2.6	586a	79.0a	501a	56998	5.0
10	18.8a	3.3	0.40	2.1	609a	75.8a	392ab	57833	5.2
20	16.1a	3.4	0.42	2.1	536a	67.0a	341b	56998	4.7
38	11.8b	3.2	0.41	2.4	375b	48.4b	278b	56486	4.5
**2010**									
0	12.7a	3.7c	0.49b	3.3	471a	63.0a	437	45609a	3.7
10	9.7b	4.1b	0.54a	3.5	392ab	53.3ab	392	48118a	3.9
20	7.0c	4.3ab	0.53a	3.4	297cb	37.2cb	249	40407b	3.8
38	5.3c	4.5a	0.55a	3.9	236c	29.4c	221	30067c	3.3
**2011**									
0	8.6a	2.8b	0.40	2.7	536	74.1	481	57213a	1.8
10	8.0a	3.3ab	0.40	2.1	609	75.8	392	57884a	1.8
20	5.9b	3.4a	0.42	2.1	536	67.0	341	52317b	1.8
38	5.0b	3.3a	0.42	2.3	425	53.4	298	49978b	1.5

† *Distance from the center of strip till row*.

‡ *Numbers followed by the same letter in a column within each year are not statistically significant at the 0.05 probability level*.

**Figure 2:**
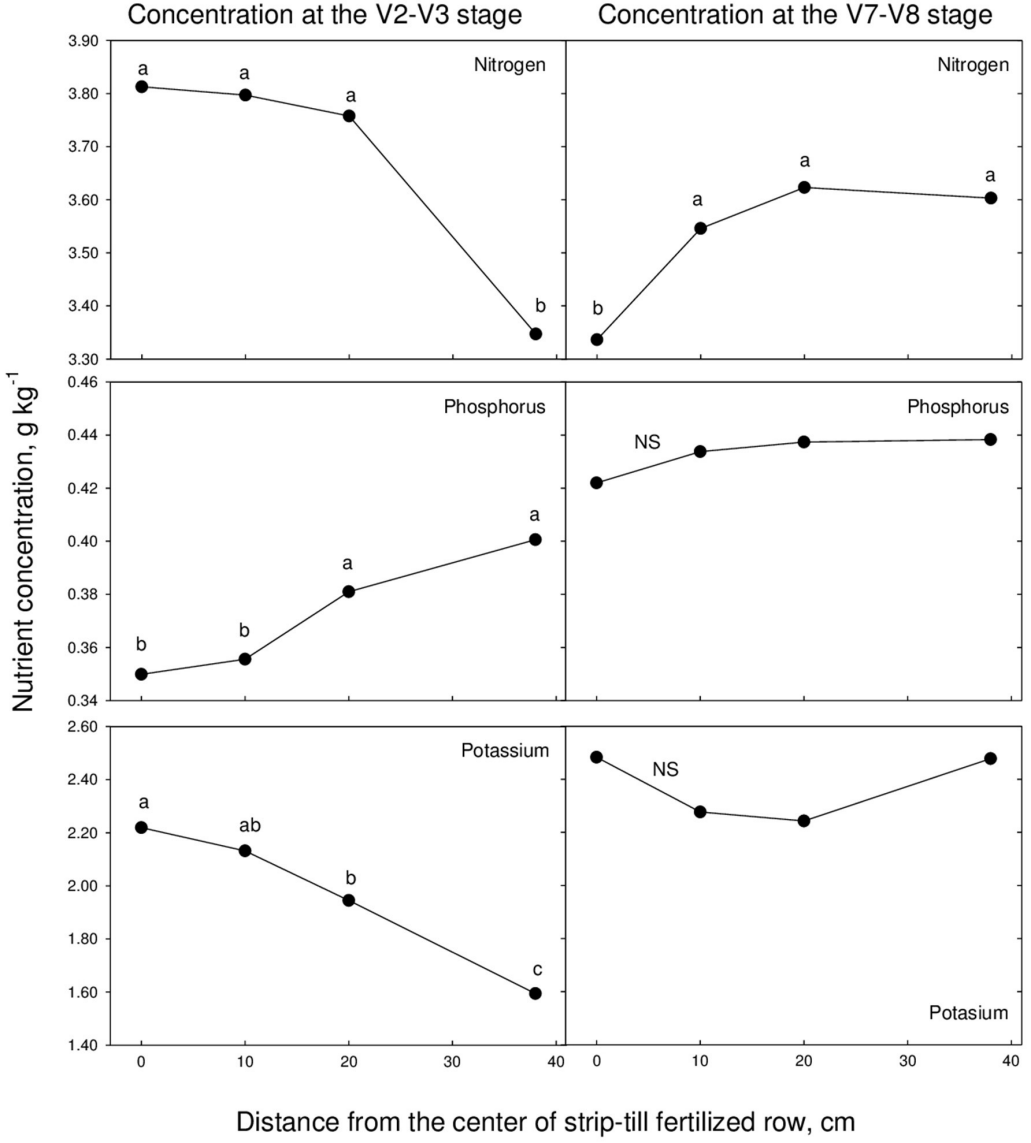
**Nitrogen, phosphorus, and potassium concentration as affected by distance from the center of strip-till fertilized row across site-years**. Samples collected at to growth stage, V2–V3 and V7–V8.

There was no consistent pattern for nutrient concentration in the plants at different planting distance from the row at the V2–V3 and V7–V8 growth stages (Tables 5, 6, Figure 2). This could be due the variability in the size of plants at different distances from the row at these growth stages. For example, a plant farther from the center of the row could have a higher concentration of nutrients but lower biomass due to cooler soil temperatures, and as a result have less total nutrient uptake. Plants may lose the potential for growth even though they have accumulated “adequate” concentrations of the limiting element (Hiatt and Massey, 1958); on the other hand when the growth-limiting element is supplied (e.g., higher soil temperatures) the relative rate of dry matter accumulation increases more rapidly than the rate of nutrient accumulation (Jarrell and Beverly, 1981).

**Figure 3:**
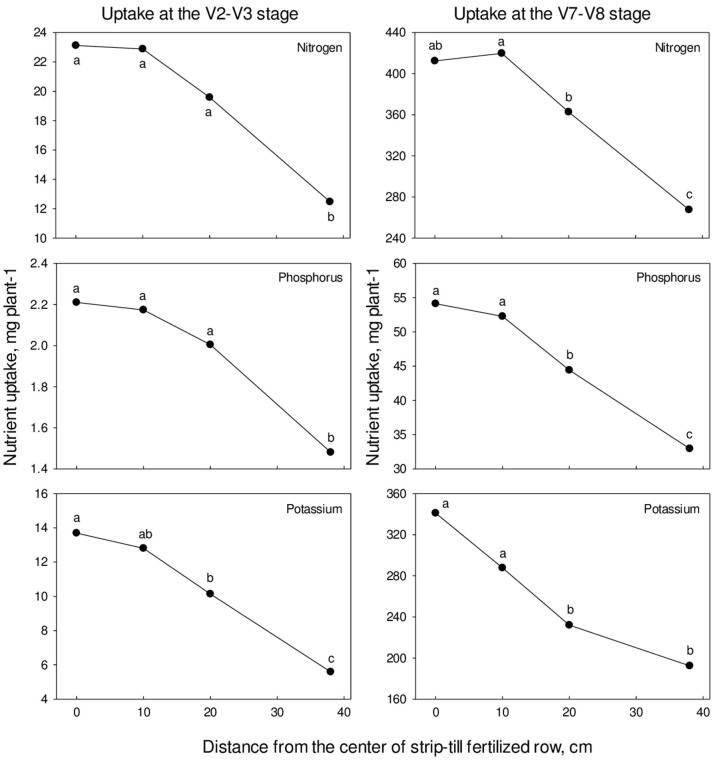
**Nitrogen, phosphorus, and potassium uptake as affected by distance from the center of strip-till fertilized row across site-years**. Samples collected at to growth stage, V2–V3 and V7–V8.

Uptake of plant nutrients (i.e., nitrogen, phosphorus, and potassium) followed a pattern similar to that for plant growth (Tables 5, 6, Figure 3). Nutrient uptake tended to be greater in plants growing closer to the band of fertilizer (Tables 5, 6, Figure 3). Nitrogen, P and K uptake was less when corn was planted 38 cm from the strip-till row (Tables 5, 6). The trend for decreasing uptake of N, P, and K by the corn plants the farther they are from the row suggest that the plants lost the beneficial effect of tillage and fertilizer placement from strip-till (Figure 3). Plants within 10 cm of the row well as those over the row showed higher plant biomass and nutrient uptake likely from the combined effect of localized tillage (strip-till) and fertilizer placement. However, this increase in early plant growth and utilization of the fertilizer applied with the strip-till did not always result in higher grain yields (Table 6). Other yield limiting factors during the growing season, such as moisture and temperature had significant role in the resulting yield and the potential benefit of planting distance from the strip-till and fertilizer band. This was particularly the case for 2006 with below average rainfall (Tables 3, 6).

**Figure 4:**
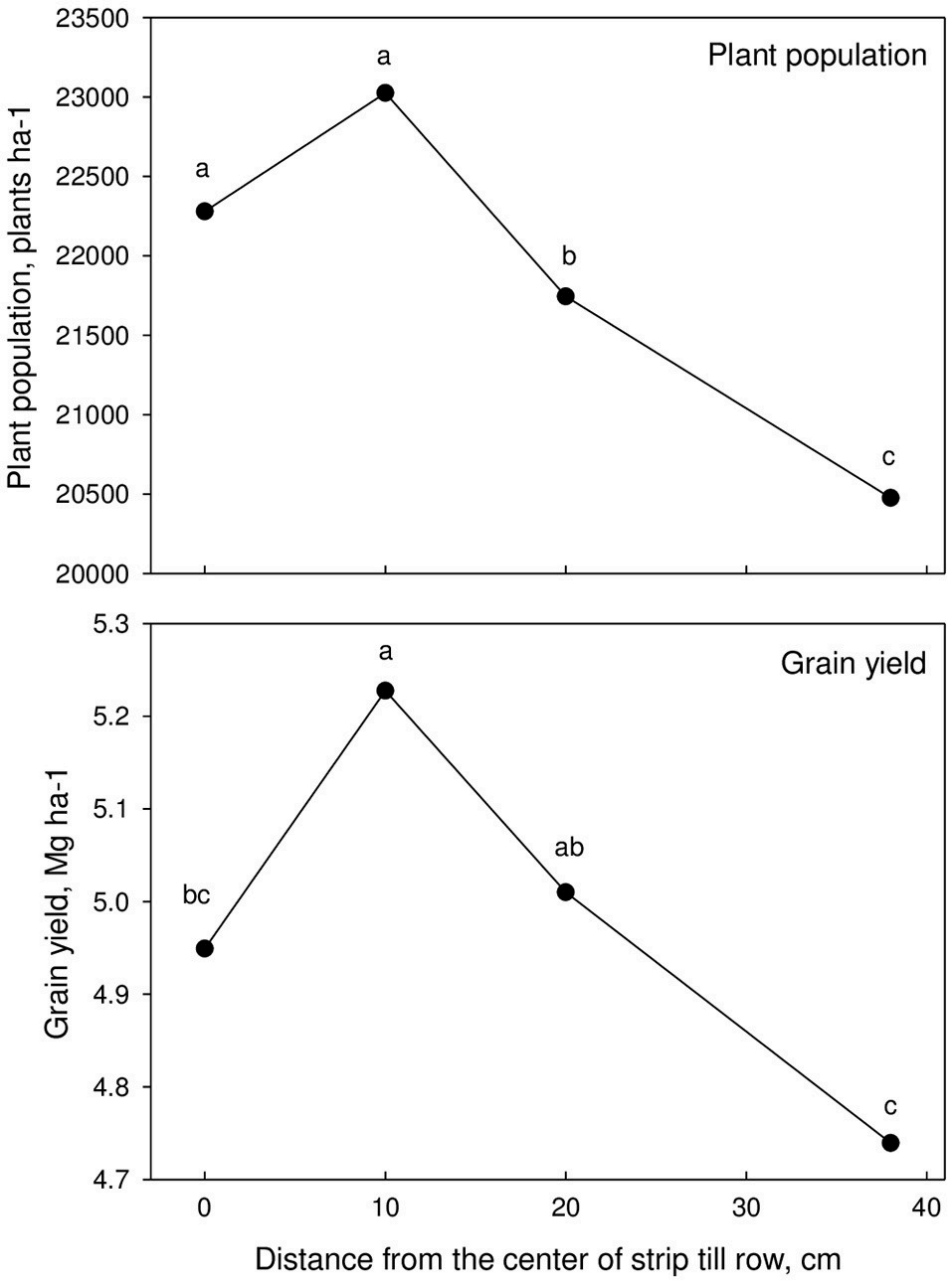
**Plant population at harvest and grain yield as affected by distance from the center of strip-till fertilized row across site-years**.

Average corn plant populations were higher when planted ~10 cm off the center of the strip-tilled fertilized rows compared with planting directly on top of the rows for every year and across years (Table 6, Figure 4). These results were statistically significant for 2 years with relatively different planting and weather conditions (2006 and 2008; Tables 2, 3). In 2006 the strip-till fertilization operation was completed 2 days before planting likely contributing to loose soils with air pockets under the row. However, in 2008 plating was completed 2 weeks after the strip-tillage operation and plant population was still higher when planted 10 cm off the center of the strip-tilled fertilized rows. Very small differences in plant populations occurred in 2009, when planting was completed 73 days after the strip-till operation. These results are similar to those of Bordoli and Mallarino (1998). They found that deep and shallow fertilizer banding did not affect plant population with any tillage treatment when applied 3–5 weeks before planting with stabilized soil with firm seedbed. In 2010, high rainfall around planting generated waterlogged and cold conditions (Tables 2, 3). This year showed the overall lowest plant population of any year of the study with significantly higher values when planting within 10 cm of the center of the row (Table 6). Low soil temperatures, a consequence of untilled soil may cause slow plant growth, and in some cases injuries and chlorosis in corn, and therefore affecting plant population (Miedema, 1982; Perez-Bidegain et al., 2007). Plant stand reduction can also occur when fungal pathogens rot seed or kill seedlings in corn when cold and wet conditions are present after planting (Nyvall, 1999). Planting 20 cm off the center of the strip-tilled fertilized rows reduced plant population 3122 plants ha-1, and 38 cm off reduced population 7308 plants ha-1 compared to planting 10 cm from the center. Early growth was reduced 38 and 53%, respectively (Tables 5, 6). Effects for 2011 were similar to 2010. Averaged across all years, the highest plant population was 10 cm off the row (Figure 4).

Across all years, the grain yield was greatest when corn was planted 10 cm off the center of the row (Figure 4). Each year had different confounded factors that contributed to the overall greatest yield with that treatment. In 2006, when corn was planted 10 cm off the center of the rows grain yield was ~8% higher than corn planted directly on top of the strip-tilled fertilized rows (Table 6). This was likely in part due to a significant reduction in plant population (1656 fewer plants ha^−1^). Results were similar in 2008 with higher corn population for treatments planted 10 cm off the center of the strip-tilled fertilized rows, this also resulted in average higher grain yield. In the 2009 the strip-till operation was completed 73 days before planting, and under these conditions there were no statically significant differences in plant population or grain yield between planting distance treatments. However, average higher grain yield was observed for treatments with planting within 10 cm of the center of the strip-till row. Yield response to planting distance from the center of strip-till was not significant in 2010 (Table 6). It is likely that overall lower plant population and excusive rainfall contributed to plant stress including nitrogen loss. In 2011, under extreme heat, drought (Campos et al., 2006), and near crop failure conditions, all treatments yielded below 1.9 Mg ha^−1^, and planting distances from the row had no significant effect on yield.

These results suggest that the best location for planting strip-tilled fertilized corn will vary depending on the growing season, condition of the strip-tilled fertilized zone, and the amount of time between planting and when the strip-tilled fertilizer operation was performed.

In a year when a strip-till operation is just prior to planting, planting 10 cm off the strip would be advisable. If the strip-till operation is well in advance of planting, and the soil has had time to settle, then planting directly over the strip or within 10 cm showed similar results. However, our results show no benefit of planting at more than 10 cm from the center of the strip-till row where the benefits of the localized tillage and fertilizer application disappeared. Strip-tilled fertilized corn should be planted in a moist, firm seedbed to obtain best stands and within 10 cm of strip-tilled fertilized rows to ensure quick contact between corn roots and fertilizer without causing potential damage to the seedlings.

Additional studies are needed to determine if these results might be different when planting strip-tilled fertilized corn on coarse-textured soils and when higher rates of fertilizer and other sources of nitrogen, such as anhydrous ammonia, are used. Phytotoxicity due to nutrients being too close to the seedling could be affected by the timing and distance of planting and more relevant for some fertilizer sources such as anhydrous ammonia.

## Conclusions

There are many benefits to planting corn into strips made in no-till fields ahead of time, but there can be disadvantages under some circumstances. Results from this study suggest that all the benefits can be obtained if planting is done within 10 cm of the center of the strip, which also reduces many of the risks associated with planting directly over the strip, such as poor or uneven stand establishment. The benefits of planting in the strips with fertilizer applied include a warmer, drier seed bed which allows for earlier planting, improved stand establishment, and early season growth than could be realized in the same field in no-till, but still maintains many of the advantages of the residue cover that no-till offers. Facilitating early season growth and development of corn in dryland production to avoid heat and drought stress at critical development stages later in the season can be very important in avoiding yield loss.

## Author contributions

Conceived, designed by KJ. Performed the experiment under field conditions: KJ and EA. Performed statistical analysis: EA, DD, FH, JK. All authors wrote and revised the paper.

## Funding

This research was partially supported by funds from Department of Agronomy and Kansas State Research and Extension. This is contribution no. 16-292-J from the Kansas Agricultural Experiment Station.

### Conflict of interest statement

The authors declare that the research was conducted in the absence of any commercial or financial relationships that could be construed as a potential conflict of interest.
